# Nasal Nitric Oxide in Children: A Review of Current Outreach in Pediatric Respiratory Medicine

**DOI:** 10.3390/children10101671

**Published:** 2023-10-09

**Authors:** Iva Mrkić Kobal, Mirjana Turkalj, Davor Plavec

**Affiliations:** 1 Outpatient Clinic for Sick Children Dr. Sabol, 10000 Zagreb, Croatia; iva.mrkic.kobal@poliklinika-sabol.hr; 2 Faculty of Medicine, JJ Strossmayer University of Osijek, 31000 Osijek, Croatia; mturkalj@bolnica-srebrnjak.hr; 3 Children’s Hospital Srebrnjak, 10000 Zagreb, Croatia; 4 Faculty of Medicine, Catholic University of Croatia, 10000 Zagreb, Croatia; 5 Prima Nova, Healthcare Institution, 10000 Zagreb, Croatia

**Keywords:** nasal nitric oxide, children, allergic rhinitis, chronic rhinosinusitis, primary ciliary dyskinesia, cystic fibrosis

## Abstract

Nasal nitric oxide (nNO) is a gas synthesized by the inducible and constitutive NO synthase (NOS) enzyme in the airway cells of the nasal mucosa. Like lung nitric oxide, it is thought to be associated with airway inflammation in various respiratory diseases in children. The aim of our review was to investigate the current state of use of nNO measurement in children. A comprehensive search was conducted using the Web of Science and PubMed databases specifically targeting publications in the English language, with the following keywords: nasal NO, children, allergic rhinitis, chronic rhinosinusitis, acute rhinosinusitis, primary ciliary dyskinesia (PCD), and cystic fibrosis (CF). We describe the use of nNO in pediatric allergic rhinitis, chronic rhinosinusitis, acute rhinosinusitis, PCD, and CF based on the latest literature. nNO is a noninvasive, clinically applicable test for use in pediatric allergic rhinitis, chronic rhinosinusitis, acute rhinosinusitis, PCD, and CF. It can be used as a complementary method in the diagnosis of these respiratory diseases and as a monitoring method for the treatment of allergic rhinitis and acute and chronic rhinosinusitis.

## 1. Introduction

Nitric oxide (NO) is a gas that can be synthesized by numerous cells of the respiratory system, including endothelial and epithelial cells, neutrophils, and alveolar macrophages. It plays roles in stimulating ciliary motility and as a mediator in inflammatory responses, has bacteriostatic and antiviral effects, and regulates bronchial muscle tone and pulmonary vascular tone [1].

Nasal nitric oxide (nNO) is a gas synthesized by the inducible and constitutive enzyme nitric oxide synthase (NOS) in the airway cells of the nasal mucosa and paranasal sinuses [2,3]. Like pulmonary nitric oxide, it has been implicated in airway inflammation in various respiratory diseases in children [4]. Measurement of nNO is a completely noninvasive procedure that can be performed in children without much effort. The preferred method for measuring nNO is chemiluminescence with a standardized procedure suitable for children 4 years of age and older and adults who can cooperate in sampling to ensure closure of the velum [5]. In preschool-aged children, electrochemical analyzers are often used instead of chemiluminescent devices. As of 2023, there is a technical standard published by the European Respiratory Society for nNO sampling in children with primary ciliary dyskinesia (PCD) [6].

Present methods for assessing nNO levels involve collecting nasal air through either direct nostril sampling (aspiration method) or during a single-breath exhalation using a nasal mask (exhalation method) [7].

Because all methods are noninvasive, nNO in children has become a research subject for diagnosis, monitoring, and evaluation of therapy for various respiratory diseases [8].

The objective of our review was to investigate the utility of nNO measurement in children diagnosed with various respiratory diseases. A comprehensive search was conducted using the Web of Science and PubMed databases specifically targeting publications in the English language, with the following keywords: nNO, children, allergic rhinitis, chronic rhinosinusitis, acute rhinosinusitis, PCD, and cystic fibrosis (CF).

## 2. nNO Role in Upper Airways

Since the identification of NO in exhaled air in 1991, extensive research has unveiled its pivotal role in the respiratory system [9]. The primary origin of intrinsic NO production within the human airway has been identified as paranasal cavities [2,3]. There are three distinct variants of NOS in the human body: neuronal, endothelial, and inducible. All three distinct variants found in the respiratory system contribute to the regulation of respiratory physiology [10]. Neuronal and endothelial NOS are considered constitutive forms, while inducible NOS was initially observed in alveolar macrophages but is now known to exist in various cell types such as alveolar cells type 2, lung fibroblasts, mastocytes, endothelial cells, and neutrophils. NO’s fundamental effects encompass neurotransmission, bronchodilation (reducing airway resistance), surfactant production, mucus secretion, and stimulation of ciliary motility, and it has an anti-inflammatory role. In the upper airway, it acts as a vasodilator and smooth muscle relaxant, stimulates mucus production, and aids in mucociliary clearance by regulating ciliary beat frequency. Furthermore, NO exhibits direct antimicrobial activity against both bacteria and viruses [1,11]. Certain research findings indicate that the body’s internal production of NO may trigger heightened activity in the submucosal glands of the respiratory system, resulting in an augmentation of mucus production [12]. Reduced NO synthesis is evident in conditions such as PCD and CF, where mucociliary clearance is impaired [13].

It remains unclear whether the decreased NO levels detected in chronic rhinosinusitis result from reduced NO production by the paranasal sinuses or from hindered NO diffusion in the nasal cavity due to sinus ostia obstruction [14].

## 3. nNO in Allergic Rhinitis

Allergic rhinitis is an atopic disease characterized by a Th-2 inflammatory response. As it represents the prevalent allergic upper respiratory condition in both children and adults, immediate efforts were made to establish a connection between nNO levels and allergic inflammation in these patients [15]. The data prior to the year 2000 were controversial because several studies claimed that nNO could be used to predict allergic rhinitis [16,17,18], whereas others, in contradiction, claimed that nNO levels in allergic rhinitis were not significantly different from those in healthy subjects [19,20]. The controversial nature of these results may be attributed to several factors, including swelling of the nasal mucosa and the blockage of sinus ostia, which can impede the distribution of nNO into the nasal cavity. Furthermore, the administration of therapeutic interventions aimed at symptom control, the timing of measurements in relation to allergen exposure, and the techniques employed for nNO measurement can all contribute to the observed discrepancies [21]. It has been demonstrated that the choice of measurement technique and the type of analyzer employed can significantly influence nNO values. Both the type of analyzer (chemiluminescence vs. electrochemical) and the aspiration rate during sampling, as well as the specific sampling technique used, can impact nNO levels. Chemiluminescence analyzers utilize exhalation against resistance and the breath-holding technique, whereas electrochemical analyzers employ the tidal breathing technique. These different approaches have been found to have varying degrees of reproducibility in measuring nNO values, with electrochemical analysis being less extensively studied and exhibiting lower reproducibility [6]. 

In 2021, Wang et al. conducted a meta-analysis of nNO levels and their clinical utility in children with allergic rhinitis, which revealed significantly elevated nNO levels compared with healthy controls [21]. Cutoff values to distinguish between AR and healthy controls were 169.4 and 161.4 nl/min in two different studies by Nesic et al. and Wen et al., respectively, with sensitivity of 83% and 100% and specificity of 80 and 94.4% [17,22].

Wang et al. also compared the influence of different types of nNO analyzers, nNO sampling methods, sampling flow rates, presence of coexisting asthma, and rhinitis symptoms, and found no evidence of differences between subgroups. The three entities that were found to undermine the differentiation of children with allergic rhinitis based on nNO values are nasal polyps, sinusitis, and marked ostial obstruction. The results are shown in Table 1 [21].

Hong and Parisi et al. demonstrated that nNO levels can also serve as a noninvasive assessment of clinical effectiveness in managing allergic rhinitis in children. They demonstrated a significant reduction in symptoms and nNO values of allergic rhinitis after treatment according to the guidelines of ARIA (nNO (91.4 ± 56.7 vs. 72.9 ± 52.4; *p* < 0.05)) and after treatment with sublingual allergen-specific immunotherapy (nNO (1035.2 ± 956.08 vs. 139.2 ± 59.01; *p* < 0.05)) [23,24]. In the study by Antosova et al., the data for treatment with H1 antihistamines alone were not reviewed. Only the combination treatment of antihistamines and nasal corticosteroids significantly decreased nNO levels in patients with allergic rhinitis [25].

In their study focusing on house dust mite-triggered allergic rhinitis, Sutiratanachai et al. observed that nNO levels could be used to discern the degree of rhinitis severity. Specifically, children with severe allergic rhinitis exhibited markedly elevated nNO levels compared to their counterparts with moderate rhinitis (as illustrated in Table 2). Fraction of exhaled NO (FeNO) levels were not changed as a function of severity of allergic rhinitis [26].

In seasonal allergic rhinitis, nNO levels are significantly elevated during and after pollen exposure, suggesting increased activity of inducible NOS during pollen exposure. The nNO levels in allergy patients were also higher during the year than in the control group [19]. In contrast to the study conducted by Sutiratanachai et al. [26], Antosova et al. did not categorize patients based on the severity of their condition. This omission suggests that individuals who are dealing with substantial mucosal swelling and nasal discharge might display reduced nNO levels, even in the presence of pronounced inflammation [25].

When examining clusters of seasonal allergic rhinitis in children, Malizia et al. found significantly different nNO values between the different clusters. Cluster I had intermediate nNO values, a lower percentage of neutrophils, low IL-5 and IL-17, high IFN-y, and IL-23 responses. Cluster 2 exhibited elevated nNO levels, an increased ocular symptom score, and a heightened IL-5 response. Cluster 3 had a neutrophil response, predominantly Th1/Th17 with significantly higher levels of IL-23, IFN-y, and IL-17 compared to other clusters which had lower nNO levels. This makes nNO a potential biomarker for endotyping allergic rhinitis [27].

## 4. nNO in Chronic Rhinosinusitis

Because the upper respiratory tract is the main source of nNO and the paranasal sinuses are the main site of nNO production [2,3], it has also been the subject of numerous studies in chronic rhinosinusitis. Chronic rhinosinusitis in children is a condition defined by a duration of at least 90 days with two or more symptoms of purulent rhinitis, nasal obstruction, facial pressure/pain, and either endoscopic evidence of mucosal edema, purulent discharge, or nasal polyposis and/or computed tomography (CT) imaging of changes in mucosa or/and in the sinuses and/or ostiomeatal complex. It is divided into two distinct entities: chronic rhinosinusitis with and without nasal polyposis [28].

Studies of nNO in chronic rhinosinusitis are mostly performed in adults [29,30,31], although there are studies in children [4].

In all studies, nNO levels were consistently decreased in chronic rhinosinusitis, especially in cases with nasal polyps [32]. Individuals with chronic rhinosinusitis and nasal polyposis demonstrate significantly lower nNO levels compared to both individuals with chronic rhinosinusitis but no nasal polyposis and healthy individuals [33].

The conclusion from the various studies is that inflammation of the nasal mucosa, especially in association with polyps, prevents the flow of nNO from the sinuses into the nasal cavities, resulting in a decrease in nNO levels [29,30,31,32]. An alternative hypothesis for the reduced nNO levels in chronic rhinosinusitis patients could be a disruption in the expression of NOS-2 synthase within the ciliary epithelial cells of the mucosa lining the paranasal sinuses [34,35].

Since nNO plays a role in airway defense, this may lead to an additional increase in the risk for recurrent infections [36].

Response to treatment of chronic rhinosinusitis is also difficult to assess. Assessing the response to therapy can involve symptom scores, endoscopic observations, and parallel measures such as saccharin clearance, as repeated use of CT examinations may not be feasible. nNO has also been shown to be a useful tool to monitor chronic rhinosinusitis response to therapy. In the study conducted by Ragab et al., they found that initial absolute nNO values exhibited an inverse correlation with changes in CT scans (Kendall’s tau-b correlation coefficient: −0.483, *p* < 0.001). Conversely, the percentage increase in nNO following both medical intervention and surgical intervention showed correlations with changes in symptom scores (Kendal’s tau-b correlation coefficient: −0.298, *p* < 0.001), saccharin clearance duration (Kendal’s tau-b correlation coefficient: −0.676, *p* < 0.001), endoscopic observations (Kendal’s tau-b correlation coefficient: −0.368, *p* < 0.001), polyp grades (Kendal’s tau-b correlation coefficient: −0.209, *p* < 0.05), and surgical scores (Kendal’s tau-b correlation coefficient: 0.291, *p* < 0.01) [31]. Absolute nNO values changed according to groups: in medically treated group the absolute nNO values increased from 724 ± 486 ppb to 1137 ± 547 ppb (*p* < 0.001), and in the surgically treated group they increased from 773 ± 426 ppb to 1129 ± 496 ppb (*p* < 0.001) at sixth months follow up [37].

## 5. nNO in Acute Rhinosinusitis in Children

Acute rhinosinusitis occurs as a complication of an upper respiratory tract infection. It is accompanied by inflammation of the mucous membrane of the paranasal sinuses and swelling of the mucous membrane, resulting in mechanical obstruction of the openings of the paranasal sinuses [38]. Difficult-to-manage cases of allergic rhinitis characterized by enduring nasal symptoms and chronic cough are frequently associated with prior episodes of acute rhinosinusitis. Nevertheless, there is limited research examining the efficacy of nNO levels as a diagnostic tool for acute bacterial rhinosinusitis in pediatric patients. Researchers conducted a comparison of nNO values among children who had unilateral maxillary sinusitis, children with allergic rhinitis, and a group of healthy controls. The nNO measurements in children diagnosed with allergic rhinitis exhibited a notable increase (765.4 ± 152.1 ppb, *p* < 0.05).

Among individuals with acute maxillary sinusitis, nNO levels on the affected side showed a significant reduction (151.2 ± 87.5 ppb, *p* < 0.05) when compared to those with allergic rhinitis and healthy participants. The nNO values on the unaffected side were not significantly lower than those in persistent allergic rhinitis and healthy subjects (748.1 ± 130.9 ppb). Following antibiotic treatment, the nNO levels on the side with the lesion rose to match those of the unaffected side, as compared to the pre-treatment values (161.4 ± 164.6 ppb vs. 9.6 ± 64.2 ppb; *p* < 0.05) [22]. Significant changes in biomarker levels and reciprocal correlations suggest that acute rhinosinusitis elicits both local and systemic inflammatory responses, with the most severe response occurring after 2 to 3 days. Of the biomarkers examined in the study by Autio et al., high-sensitivity C-reactive protein (CRP) and nNO most accurately reflect this inflammatory response, with CRP levels increasing and nNO levels decreasing in parallel with the increase in inflammation level [34].

## 6. nNO in Primary Ciliary Dyskinesia

PCD is a rare hereditary disorder inherited in an autosomal recessive manner, characterized by the absence of ciliary structure or a deficiency in its function. It affects approximately 1 in 7500 individuals worldwide [39]. Because the disorder impairs ciliary function, abnormal mucus excretion from the upper and lower airways occurs, resulting in respiratory distress in neonates of unknown etiology, productive wet cough in infancy, perennial rhinosinusitis, chronic bronchitis, and bronchiectasis. The discovery of reduced nNO levels in individuals with PCD was made over two decades ago. When nNO levels are observed in children with PCD, they are extreme, and even less than 10 times lower than normal levels in healthy patients [40]. Although nNO levels are quite low in PCD, similar nNO levels are reported in cystic fibrosis (CF) [41], diffuse panbronchiolitis [42], and acute viral respiratory infection [43]. For a correct differential diagnosis, it is important to know that patients with allergic rhinitis and asthma, chronic obstructive pulmonary disease, primary immunodeficiency, non-CF-bronchiectasis, and chronic rhinosinusitis without nasal polyposis have higher nNO levels than patients with PCD (Table 3) [5]. The situation is somewhat different in infants and young children, where nNO levels can often overlap with those of healthy infants. These levels in infants may be influenced by external factors, such as high concentrations of the environment NO [43,44]. Even in children 2 to 5 years of age, nNO cannot always be used to distinguish between healthy individuals and PCD patients. nNO levels increase rapidly during the first 18 months and then gradually approach adult levels at 12 years of age [44,45] (Table 4). Because there are few standards for nNO in neonates, healthy infants, and young children, measurement of nNO in these age groups is limited to research centers or, for ages older than 12 months, to experienced technicians in centers specializing in PCD [6].

Clinical studies in children 5 years of age and older with PCD have demonstrated high accuracy of nNO measurement [46]. As demonstrated by Leigh et al. in patients with suspected PCD, nNO < 77 ppb, when measured with a chemiluminescence NO analyzer while blowing into a mouth resistor, has a specificity and sensitivity greater than 95% for the diagnosis of PCD compared with electronic microscopy and/or genetic testing [40]. Only chemiluminescence technology has been rigorously validated in clinical trials for the detection of PCD [46,47]. Although some small studies on electrochemical NO analyzers have been performed for nNO measurement in PCD, they are not recommended for nNO measurement in PCD [48,49].

It is also very important that patients have a high pretest probability for PCD before nNO testing is performed, because nNO measurement in the general population results in poorer diagnostic accuracy. In a study involving a population with a high likelihood of PCD, the test exhibited a sensitivity of 93.6% (95% CI 78.5% to 99.0%) and a specificity of 84.1% (95% CI 78.9% to 88.4%). The positive predictive value was calculated to be 46% (95% CI 30.2% to 54.5%), while the negative predictive value stood at 99.1% (95% CI 96.6% to 99.9%) [50]. To improve diagnostic accuracy, it is strongly recommended that the nNO test be repeated in two separate examinations at least two weeks apart [5]. The nNO levels in patients with PCD remain low over time [40].

## 7. nNO in Cystic Fibrosis

Cystic fibrosis (CF) is a hereditary genetic disorder passed down through an autosomal recessive pattern. It arises due to a variety of mutations occurring within the CFTR gene, which stands for the CF transmembrane conductance regulator gene. This gene is responsible for encoding a channel that allows the movement of chloride and bicarbonate ions. Individuals with mutation can have a variety of medical conditions that affect the respiratory, endocrine, gastrointestinal, pancreatic, biliary, and reproductive systems [51].

Within the domain of respiratory afflictions, children affected with CF confront the exigencies of enduring chronic bronchitis concomitant with bronchiectasis, intractable rhinosinusitis, and iterative ear infections. The quantification of nNO in the context of CF reveals levels that are understandably diminished compared to those in typically healthy children. However, these levels still register lower than those observed in cases of chronic rhinosinusitis, albeit surpassing those encountered in PCD. Güney et al. conducted a study to establish reference values for nNO using a chemiluminescence analyzer during nasal quiet exhalation in children with PCD or CF, and healthy children. They observed that the mean nNO values were 10.4 ± 8.3 ppb, 22.8 ± 18.7 ppb, and 21 ± 8.9 ppb, respectively. Notably, nNO levels in children with PCD were found to be significantly lower than those in children with CF and the healthy control group (*p* < 0.05) [52].

## 8. Discussion

The assessment of nasal nitric oxide (nNO) remains, in part, a research modality characterized by the absence of established normative benchmarks and uniform protocols tailored to distinct age cohorts and diverse analyzers, considering the array of respiratory maneuvers employed. Since paranasal sinuses show age-dependent development, and they are the most important source of nNO, we still need to obtain additional normative data for young children and infants, electrochemical analyzers, and tidal breathing techniques. Moreover, the outcomes of nNO measurements can be subject to the impact of environmental variables concerning the environmental level of NO, thereby circumscribing the practicability of this method to specialized facilities. The increase in studies regarding management of high ambient NO levels could be useful. nNO is also influenced by factors such as nasal airflow, humidity, and temperature. Dietary intake of nitrate-rich food and exposure to environmental factors such as pollution can influence nNO levels. These factors should be controlled for in research studies and clinical assessments. Some medications, such as nasal corticosteroids, can affect nNO measurements. Patients’ medication histories should be taken into account when interpreting nNO results. Additionally, anatomical abnormalities or blockages in nasal passages can alter nNO levels.

Further research will hopefully improve the clinical application of nNO measurement in everyday practice, but current knowledge can be used for the benefit of patients as nNO is a completely noninvasive method. It is important to emphasize that interpretation of nNO levels should be performed in conjunction with clinical findings and other diagnostic tests. Elevated or decreased nNO levels may indicate airway inflammation, but the underlying cause requires further investigation.

## 9. Conclusions

nNO is a noninvasive, clinically applicable test for use in pediatric allergic rhinitis, chronic rhinosinusitis, acute rhinosinusitis, primary ciliary dyskinesia, and cystic fibrosis. It can be used as a complementary method in the diagnosis of these respiratory diseases and as a monitoring tool during the treatment of allergic rhinitis and acute and chronic rhinosinusitis.

## Figures and Tables

**Table 1:** Subgroup analysis of nNO in allergic rhinitis patients.

Subgroup Analysis	Effect Size SMD (95% CI) in nNO
Studies of different kind of analyzers Stationary Handheld	SMD: 0.554; 95%CI: 0.260, 0.849; *p* = 0.010; I^2^ = 46.3%, *p* = 0.114 SMD: 1.526; 95%CI: 0.361, 2.691; *p* = 0.010; I^2^ = 95.6%, *p* = 0.000
Studies of different sampling techniques BH ER	SMD: 1.717; 95%CI: 0.029, 3.404; *p* = 0.046; I^2^ = 97%, *p* = 0.000 SMD: 0.638; 95%CI: 0.337, 0.938; *p* = 0.000; I^2^ = 57.3%, *p* = 0.039
Studies of different sampling flow rates 3 L/min 0.3 L/min	SMD: 0.374; 95%CI: 0.092, 0.656; *p* = 0.009; I^2^ = 0.0%, *p* = 0.369 SMD: 1.316; 95%CI: 0.368, 2.264; *p* = 0.007; I^2^ = 95.3%, *p* = 0.000
Studies of the AR patients with/without asthma With asthma Without asthma	SMD: 1.011; 95%CI: 0.593, 1.428; *p* = 0.000; I^2^ = not applicable, *p* = not applicable SMD: 0.987; 95%CI: 0.310, 1.665; *p* = 0.004; I^2^ = 93.6%, *p* = 0.000
Studies of the AR patients with/without rhinitis symptoms Having rhinitis symptoms Not sure having rhinitis symptoms	SMD: 0.404; 95%CI: 0.169, 0.638; *p* = 0.001; I^2^ = 0.0%, *p* = 0.545 SMD: 1.438; 95%CI: 0.529, 2.346; *p* = 0.002; I^2^ = 94.5%, *p* = 0.000
Studies of AR patients with/without nasal polyps With nasal polyps Without nasal polyps	SMD: −0.215; 95%CI: −0.905, 0.476; *p* = 0.543; I^2^ = not applicable, *p* = not applicable SMD: 1.195; 95%CI: 0.200, 2.189; *p* = 0.019; I^2^ = 96%, *p* = 0.000
Studies of AR patients with/without sinusitis With sinusitis Without sinusitis	SMD: 0.972; 95%CI: −3.627, 5.571; *p* = 0.679; I^2^ = 99.3%, *p* = 0.000 SMD: 1.102; 95%CI: 0.689, 1.515; *p* = 0.000; I^2^ = 70.5%, *p* = 0.009
Studies of AR patients excluding/not excluding smoking Excluding smoking Not excluding smoking	SMD: 0.723; 95%CI: 0.174, 1.272; *p* = 0.010; I^2^ = 67.2%, *p* = 0.027 SMD: 1.157; 95%CI: 0.264, 2.049; *p* = 0.011; I^2^ = 95.8%, *p* = 0.000
Studies of AR patients with/without marked ostial obstruction With marked ostial obstruction Without marked ostial obstruction	SMD: 0.723; 95%CI: 0.174, 1.272; *p* = 0.010; I^2^ = 67.2%, *p* = 0.027 SMD: 1.157; 95%CI: 0.264, 2.049; *p* = 0.011; I^2^ = 95.8%, *p* = 0.000

Abbreviations: AR, allergic rhinitis; BH, breath hold; CI, confidence interval; ER exhalation against resistance; nNO, nasal nitric oxide; SMD, standardized mean differences. Modified from Wang et al. [21].

**Table 2:** nNO levels in moderate and severe HDM-induced allergic rhinitis.

	Moderate Allergic Rhinitis	Severe Allergic Rhinitis
nNO levels (ppb)	941.3	1652.05
*p*-value		0.002
Cut -off Value for nNO (ppb)		1350
AUC (95%CI)		0.764 (0.616–0.911)
Sensitivity (%)		78
Specificity (%)		71

Modified from Sutiratanachai et al. [26].

**Table 3:** nNO values in different respiratory diseases.

Nasal NO Values (ppb)				
Healthy Controls	Primary Ciliary Dyskinesia	Cystic Fibrosis	Chronic Rhinosinusitis without Nasal Polyposis	Non-CF-Bronchiectasis
543–976	17–180	241–896	862–3601	516–1098

**Table 4:** nNO values according to age.

Newborn	46 ppb	IQR 29–69 ppb
AGE 2	238 ppb	IQR 203–389 ppb
AGE 4–5	283.5 ppb	SD +/− 107.4 ppb
AGE 5–6	294.3 ppb	SD +/− 121.6 ppb
AGE > 6	350.2 ppb	SD +/− 123 ppb
AGE 6–17	449 ppb	SD +/− 115 ppb

Rate of increase from newborn to age 2 is 5.4% monthly. Modified from Piacentini GL, et al. [45].

## References

[1] Ricciardolo F.L.M. (2003). Multiple roles of nitric oxide in the airways. Thorax.

[2] Jorissen M., Lefevere L., Willems T. (2001). Nasal nitric oxide. Allergy.

[3] Kawasumi T., Takeno S., Ishikawa C., Takahara D., Taruya T., Takemoto K., Hamamoto T., Ishino T., Ueda T. (2021). The Functional Diversity of Nitric Oxide Synthase Isoforms in Human Nose and Paranasal Sinuses: Contrasting Pathophysiological Aspects in Nasal Allergy and Chronic Rhinosinusitis. Int. J. Mol. Sci..

[4] Ferraro V.A., Zanconato S., Baraldi E., Carraro S. (2019). Nitric Oxide and Biological Mediators in Pediatric Chronic Rhinosinusitis and Asthma. J. Clin. Med..

[5] Shapiro A.J., Dell S.D., Gaston B., O’connor M., Marozkina N., Manion M., Hazucha M.J., Leigh M.W. (2020). Nasal Nitric Oxide Measurement in Primary Ciliary Dyskinesia. A Technical Paper on Standardized Testing Protocols. Ann. Am. Thorac. Soc..

[6] Beydon N., Kouis P., Marthin J.K., Latzin P., Colas M., Davis S.D., Haarman E., Harris A.L., Hogg C., Kilbride E. (2023). Nasal nitric oxide measurement in children for the diagnosis of primary ciliary dyskinesia: European Respiratory Society technical standard. Eur. Respir. J..

[7] Ambrosino P., Parrella P., Formisano R., Papa A., Spedicato G.A., Di Minno M.N.D., Motta A., Maniscalco M. (2020). Clinical application of nasal nitric oxide measurement in allergic rhinitis. Ann. Allergy Asthma Immunol..

[8] Pappalardo M.G., Parisi G.F., Tardino L., Savasta S., Brambilla I., Marseglia G.L., Licari A., Leonardi S. (2019). Measurement of nitric oxide and assessment of airway diseases in children: An update. Minerva Pediatr..

[9] Ricciardolo F.L.M., Sterk P.J., Gaston B., Folkerts G., Marozkina N., Bosch J., Cotton C., Smith L., Seckler J., Zaman K. (2004). Nitric Oxide in Health and Disease of the Respiratory System. Physiol. Rev..

[10] Förstermann U., Sessa W.C. (2012). Nitric oxide synthases: Regulation and function. Eur. Heart J..

[11] Antosova M., Mokra D., Pepucha L., Plevkova J., Buday T., Sterusky M., Bencova A. (2017). Physiology of Nitric Oxide in the Respiratory System. Physiol. Res..

[12] Soodaeva S., Kubysheva N., Klimanov I., Nikitina L., Batyrshin I. (2019). Features of Oxidative and Nitrosative Metabolism in Lung Diseases. Oxidative Med. Cell. Longev..

[13] Eggenkemper L., Schlegtendal A., Maier C., Lücke T., Brinkmann F., Beckmann B., Tsikas D., Koerner-Rettberg C. (2023). Impaired Nitric Oxide Synthetase Activity in Primary Ciliary Dyskinesia—Data-Driven Hypothesis. J. Clin. Med..

[14] Yoshida K., Takabayashi T., Imoto Y., Sakashita M., Narita N., Fujieda S. (2018). Reduced nasal nitric oxide levels in patients with eosinophilic chronic rhinosinusitis. Allergol. Int..

[15] Tenero L., Vaia R., Ferrante G., Maule M., Venditto L., Piacentini G., Senna G., Caminati M. (2023). Diagnosis and Management of Allergic Rhinitis in Asthmatic Children. J. Asthma Allergy.

[16] Lee K.J., Cho S.H., Lee S.H., Tae K., Yoon H.J., Kim S.H., Jeong J.H. (2012). Nasal and Exhaled Nitric Oxide in Allergic Rhinitis. Clin. Exp. Otorhinolaryngol..

[17] Nesic V.S., Djordjevic V.Z., Tomic-Spiric V., Dudvarski Z.R., A Soldatovic I., A Arsovic N. (2016). Measuring nasal nitric oxide in allergic rhinitis patients. J. Laryngol. Otol..

[18] Wang P.-P., Wang G.-X., Ge W.-T., Tang L.-X., Zhang J., Ni X. (2017). Nasal nitric oxide in allergic rhinitis in children and its relationship to severity and treatment. Allergy Asthma Clin. Immunol..

[19] Moody A., Fergusson W., Wells A., Bartley J., Kolbe J. (2006). Nasal levels of nitric oxide as an outcome variable in allergic upper respiratory tract disease: Influence of atopy and hayfever on nNO. Am. J. Rhinol..

[20] Henriksen A., Sue-Chu M., Holmen T.L., Langhammer A., Bjermer L. (1999). Exhaled and nasal NO levels in allergic rhinitis: Relation to sensitization, pollen season and bronchial hyperresponsiveness. Eur. Respir. J..

[21] Wang B., Wu Z., Wang F., Yin Z., Shi L., Liu Y. (2021). Nasal nitric oxide testing for allergic rhinitis patients: Systematic review and meta-analysis. Immun. Inflamm. Dis..

[22] Wen Y.-S., Lin C.-Y., Yang K.D., Hung C.-H., Chang Y.-J., Tsai Y.-G. (2019). Nasal nitric oxide is a useful biomarker for acute unilateral maxillary sinusitis in pediatric allergic rhinitis: A prospective observational cohort study. World Allergy Organ. J..

[23] Hong S., Jo C.-G., Kim H., Lee Y.-S., Bae W.Y., Jung J.-A. (2022). Changes in levels of fractional exhaled and nasal nitric oxide after treatment in allergic rhinitis. Allergy Asthma Respir. Dis..

[24] Parisi G.F., Manti S., Papale M., Amato M., Licari A., Marseglia G.L., Leonardi S. (2021). Nasal Nitric Oxide and Nasal Cytology as Predictive Markers of Short-Term Sublingual Allergen-Specific Immunotherapy Efficacy in Children with Allergic Rhinitis. Am. J. Rhinol. Allergy.

[25] Antosova M., Bencova A., Mokra D., Plevkova J., Pepucha L., Buday T. (2020). Exhaled and Nasal Nitric Oxide—Impact for Allergic Rhinitis. Physiol. Res..

[26] Sutiratanachai W., Kanchongkittiphon W., Klangkalya N., Jotikasthira W., Kiewngam P., Manuyakorn W. (2022). Airway Nitric Oxide in Children with HDM-Induced Allergic Rhinitis. Am. J. Rhinol. Allergy.

[27] Malizia V., Ferrante G., Cilluffo G., Gagliardo R., Landi M., Montalbano L., Fasola S., Profita M., Licari A., Marseglia G.L. (2022). Endotyping Seasonal Allergic Rhinitis in Children: A Cluster Analysis. Front. Med..

[28] Brietzke S.E., Shin J.J., Choi S., Lee J.T., Parikh S.R., Pena M., Prager J.D., Ramadan H., Veling M., Corrigan M. (2014). Clinical Consensus Statement: Pediatric Chronic Rhinosinusitis. Otolaryngol. Neck Surg..

[29] Frendø M., Håkansson K., Schwer S., Ravn A.T., Meteran H., Porsbjerg C., Backer V., von Buchwald C. (2018). Exhaled and nasal nitric oxide in chronic rhinosinusitis patients with nasal polyps in primary care. Rhinol. J..

[30] Maniscalco M. (2017). Nasal nitric oxide as biomarker in the evaluation and management of chronic rhino-sinusitis with nasal polyposis. Eur. Arch. Oto-Rhino-Laryngol..

[31] Fu C.-H., Tseng H.-J., Huang C.-C., Chang P.-H., Chen Y.-W., Lee T.-J. (2017). Nasal nitric oxide in unilateral sinus disease. PLoS ONE.

[32] Benedict J.J., Lelegren M., Han J.K., Lam K. (2023). Nasal Nitric Oxide as a Biomarker in the Diagnosis and Treatment of Sinonasal Inflammatory Diseases: A Review of the Literature. Ann. Otol. Rhinol. Laryngol..

[33] Ambrosino P., Molino A., Spedicato G.A., Parrella P., Formisano R., Motta A., Di Minno M.N.D., Maniscalco M. (2020). Nasal Nitric Oxide in Chronic Rhinosinusitis with or without Nasal Polyps: A Systematic Review with Meta-Analysis. J. Clin. Med..

[34] Autio T.J., Koskenkorva T., Leino T.K., Koivunen P., Alho O.-P. (2017). Longitudinal analysis of inflammatory biomarkers during acute rhinosinusitis. Laryngoscope.

[35] Asano T., Takemura M., Kanemitsu Y., Yokota M., Fukumitsu K., Takeda N., Ichikawa H., Hijikata H., Uemura T., Takakuwa O. (2018). Combined measurements of fractional exhaled nitric oxide and nasal nitric oxide levels for assessing upper airway diseases in asthmatic patients. J. Asthma.

[36] Rouby J.-J. (2003). The nose, nitric oxide, and paranasal sinuses: The outpost of pulmonary antiinfectious defenses?. Am. J. Respir. Crit. Care Med..

[37] Ragab S.M., Lund V.J., Saleh H.A., Scadding G. (2006). Nasal nitric oxide in objective evaluation of chronic rhinosinusitis therapy. Allergy.

[38] Wald E.R., Applegate K.E., Bordley C., Darrow D.H., Glode M.P., Marcy S.M., Nelson C.E., Rosenfeld R.M., Shaikh N., Smith M.J. (2013). Clinical Practice Guideline for the Diagnosis and Management of Acute Bacterial Sinusitis in Children Aged 1 to 18 Years. Pediatrics.

[39] Hannah W.B., A Seifert B., Truty R., A Zariwala M., Ameel K., Zhao Y., Nykamp K., Gaston B. (2022). The global prevalence and ethnic heterogeneity of primary ciliary dyskinesia gene variants: A genetic database analysis. Lancet Respir. Med..

[40] Leigh M.W., Hazucha M.J., Chawla K.K., Baker B.R., Shapiro A.J., Brown D.E., LaVange L.M., Horton B.J., Qaqish B., Carson J.L. (2013). Standardizing Nasal Nitric Oxide Measurement as a Test for Primary Ciliary Dyskinesia. Ann. Am. Thorac. Soc..

[41] Noone P.G., Leigh M.W., Sannuti A., Minnix S.L., Carson J.L., Hazucha M., Zariwala M.A., Knowles M.R. (2004). Primary ciliary dyskinesia: Diagnostic and phenotypic features. Am. J. Respir. Crit. Care Med..

[42] Nakano H., Ide H., Imada M., Osanai S., Takahashi T., Kikuchi K., Iwamoto J. (2000). Reduced Nasal Nitric Oxide in Diffuse Panbronchiolitis. Am. J. Respir. Crit. Care Med..

[43] Marthin J.K., Philipsen M.C., Rosthoj S., Nielsen K.G. (2018). Infant nasal nitric oxide over time: Natural evolution and impact of respiratory tract infection. Eur. Respir. J..

[44] Adams P.S., Tian X., Zahid M., Khalifa O., Leatherbury L., Lo C.W. (2015). Establishing normative nasal nitric oxide values in infants. Respir. Med..

[45] Piacentini G.L., Bodini A., Peroni D.G., Sandri M., Brunelli M., Pigozzi R., Boner A.L. (2010). Nasal nitric oxide levels in healthy pre-school children. Pediatr. Allergy Immunol..

[46] Shapiro A.J., Josephson M., Rosenfeld M., Yilmaz O., Davis S.D., Polineni D., Guadagno E., Leigh M.W., Lavergne V. (2017). Accuracy of Nasal Nitric Oxide Measurement as a Diagnostic Test for Primary Ciliary Dyskinesia. A Systematic Review and Meta-analysis. Ann. Am. Thorac. Soc..

[47] Shapiro A.J., Zariwala M.A., Ferkol T.W., Davis S.D., Sagel S.D., Dell S.D., Rosenfeld M., Olivier K.N., Milla C., Daniel S.J. (2016). Diagnosis, monitoring, and treatment of primary ciliary dyskinesia: PCD foundation consensus recommendations based on state of the art review. Pediatr. Pulmonol..

[48] Harris A., Bhullar E., Gove K., Joslin R., Pelling J., Evans H.J., Walker W.T., Lucas J.S. (2014). Validation of a portable nitric oxide analyzer for screening in primary ciliary dyskinesias. BMC Pulm. Med..

[49] Marthin J.K., Nielsen K.G. (2013). Hand-Held Tidal Breathing Nasal Nitric Oxide Measurement—A Promising Targeted Case-Finding Tool for the Diagnosis of Primary Ciliary Dyskinesia. PLoS ONE.

[50] A Collins S., Behan L., Harris A., Gove K., Lucas J.S. (2016). The dangers of widespread nitric oxide screening for primary ciliary dyskinesia. Thorax.

[51] Polgreen P.M., Comellas A.P. (2022). Clinical Phenotypes of Cystic Fibrosis Carriers. Annu. Rev. Med..

[52] Güney E., Emiralioğlu N., Cinel G., Yalçın E., Doğru D., Kiper N., Özçelik H.U. (2019). Nasal nitric oxide levels in primary ciliary dyskinesia, cystic fibrosis and healthy children. Turk. J. Pediatr..

